# Biallelic variants of *ATP13A3* cause dose-dependent childhood-onset pulmonary arterial hypertension characterised by extreme morbidity and mortality

**DOI:** 10.1136/jmedgenet-2021-107831

**Published:** 2021-09-07

**Authors:** Rajiv D Machado, Carrie L Welch, Matthias Haimel, Marta Bleda, Elizabeth Colglazier, John D Coulson, Marusa Debeljak, Josef Ekstein, Jeffrey R Fineman, William Christopher Golden, Emily L Griffin, Charaka Hadinnapola, Michael A Harris, Yoel Hirsch, Julie Elizabeth Hoover-Fong, Lawrence Nogee, Lewis H Romer, Samo Vesel, NIHR Bioresource – Rare Diseases, Stefan Gräf, Nicholas W Morrell, Laura Southgate, Wendy K Chung

**Affiliations:** 1 Molecular and Clinical Sciences Research Institute, St George's University of London, London, UK; 2 Department of Pediatrics, Columbia University Irving Medical Center, New York, New York, USA; 3 NIHR Bioresource – Rare Diseases, University of Cambridge, Cambridge, Cambridgeshire, UK; 4 Department of Medicine, University of Cambridge School of Clinical Medicine, Cambridge, Cambridgeshire, UK; 5 Department of Nursing, University of California San Francisco, San Francisco, California, USA; 6 Department of Pediatrics, Johns Hopkins University School of Medicine, Baltimore, Maryland, USA; 7 Clinical Institute of Special Laboratory Diagnostics, University Medical Centre Ljubljana, University Children's Hospital, Ljubljana, Slovenia; 8 Faculty of Medicine, Institute of Cell Biology, University of Ljubljana, Ljubljana, Slovenia; 9 Dor Yeshorim, Committee for Prevention of Jewish Genetic Diseases, Brooklyn, New York, USA; 10 Department of Pediatrics and Cardiovascular Research Institute, University of California San Francisco, San Francisco, California, USA; 11 Vici Syndrome Foundation, Inc, Silver Spring, Maryland, USA; 12 Department of Genetic Medicine, Johns Hopkins University School of Medicine, Baltimore, Maryland, USA; 13 Department of Anesthesiology and Critical Care Medicine, Cell Biology, Biomedical Engineering, and the Center for Cell Dynamics, Johns Hopkins University School of Medicine, Baltimore, Maryland, USA; 14 Department of Cardiology, University Medical Centre Ljubljana, University Children's Hospital, Ljubljana, Slovenia; 15 Department of Paediatrics, Teaching Hospital Celje, Celje, Slovenia; 16 Department of Medicine, Columbia University Irving Medical Center, New York, NY, USA

**Keywords:** pediatrics, genetics, pulmonary heart disease

## Abstract

**Background:**

The molecular genetic basis of pulmonary arterial hypertension (PAH) is heterogeneous, with at least 26 genes displaying putative evidence for disease causality. Heterozygous variants in the *ATP13A3* gene were recently identified as a new cause of adult-onset PAH. However, the contribution of *ATP13A3* risk alleles to child-onset PAH remains largely unexplored.

**Methods and results:**

We report three families with a novel, autosomal recessive form of childhood-onset PAH due to biallelic *ATP13A3* variants. Disease onset ranged from birth to 2.5 years and was characterised by high mortality. Using genome sequencing of parent–offspring trios, we identified a homozygous missense variant in one case, which was subsequently confirmed to cosegregate with disease in an affected sibling. Independently, compound heterozygous variants in *ATP13A3* were identified in two affected siblings and in an unrelated third family. The variants included three loss of function variants (two frameshift, one nonsense) and two highly conserved missense substitutions located in the catalytic phosphorylation domain. The children were largely refractory to treatment and four died in early childhood. All parents were heterozygous for the variants and asymptomatic.

**Conclusion:**

Our findings support biallelic predicted deleterious *ATP13A3* variants in autosomal recessive, childhood-onset PAH, indicating likely semidominant dose-dependent inheritance for this gene.

## Background

Pulmonary arterial hypertension (PAH (MIM: 178 600)) is a disease of complex aetiology, clinically characterised by incomplete penetrance, sex bias and variable age of onset, both within and between families. While most families exhibit autosomal dominant transmission, rare examples of autosomal recessive inheritance have been observed.^1 2^ The major genetic risk factor for both familial (ie, heritable PAH (HPAH)) and idiopathic PAH (IPAH) is haploinsufficiency of the bone morphogenetic protein receptor 2 gene (*BMPR2*).^1 2^ However, approximately 80% of IPAH cases remain genetically undiagnosed. Recent sequencing studies have accelerated the identification of additional risk genes,^3–5^ including monoallelic *ATP13A3* variants associated with adult-onset IPAH across genetic ancestries.^3 4 6 7^ *ATP13A3* encodes a P-type ATPase, a cation transporter with a documented role in the polyamine transport system.^8^

Compared with adult-onset disease, childhood-onset PAH (cPAH) is marked by high morbidity, resistance to available therapies and poor survival.^9^ Emerging data also indicate a strikingly different genomic architecture and distribution of genetic variants.^4 9 10^ De novo variants cause ~15% of cPAH. Monoallelic heritable variants in *BMPR2*, *TBX4*, *ABCC8* and *GDF2* likely cause 2%–6% of HPAH/IPAH in children, while variants in *KCNK3*, *ACVRL1*, *ENG* and *SMAD9* are rare causes of cPAH.^9^ Biallelic variants in *GDF2* and *KCNK3* have been identified in very early-onset, severe cPAH in at least two families.^11 12^ Herein, we identified biallelic variants in *ATP13A3* as the genetic cause of cPAH in three families including five affected children presenting under 3 years of age.

## Patient cohort

### Family 1

Proband II.1 presented with right heart failure at age 2.5 years. Echocardiography established right ventricular (RV) dilatation and hypertrophy with right atrial enlargement. Right heart catheterisation (RHC) was deemed too risky and was not performed. The child was diagnosed with IPAH, treated with sildenafil and diuretics, but died at 4 years of age due to right heart failure. The brother of the proband, II.2, was monitored from birth. At 2.5 years of age, echocardiography revealed signs of suprasystemic pressure in the right ventricle with evidence of hypertrophy and dilation. There was also right-to-left shunting through a foramen ovale with percutaneous saturation of ~90%. RHC showed an elevated mean pulmonary artery pressure (mPAP) of 40 mm Hg. Despite triple therapy (dual oral bosentan and sildenafil supplemented with epoprostenol after 15 months), the patient required lung transplantation at age 4.5 years. He died at age 8.5 years after contracting pneumonia. Both parents (I.1 and I.2) and a sister (II.3) remain healthy with no signs of PAH. The family is of Slovenian descent (table 1).

**Table 1:** Clinical phenotypes and *ATP13A3* variants identified in childhood-onset, autosomal recessive PAH

Case	Sex	Age at diagnosis	Age at death	mPAP (mm Hg)	PVRi (Woods units)	Treatment	Variant(s)	Mutation type	MAF gnomAD v2.1.1 (controls)	CADD score h38v1.6	SIFT	PolyPhen2 (HumVar)	Previously-reported for PAH?	ACMG class
F1 II.1	M	2.5 years	4 years	nk		Sildenafil and diuretics	c.2563G>A (p.Val855Met) rs1489314131	Missense (homozygous)	absent	26.5	D	D	Barozzi 2019^14^	LP
F1 II.2	M	2.5 years	8 years	40		Sildenafil, bosentan, intravenous epoprostenol and lung transplantation	c.2563G>A (p.Val855Met) rs1489314131	Missense (homozygous)	absent	26.5	D	D	Barozzi 2019^14^	LP
F2 II.2	M	5 months	11 months	34*	11.8	Oxygen, diuretics, sildenafil, bosentan and intravenous treprostinil	c.2549dupT (p.Met850Ilefs13) rs1560082927	Frameshift	9.21e-6 (no homo)	33	D	D	Zhu 2019^4^	P
							c.2227C>T (p.Arg743Cys)	Missense	absent	32	D	D	Zhu 2019^4^	LP
F2 II.4	F	7 days	17 months	51*	11.8	Oxygen, diuretics, sildenafil, bosentan and subcutaneous treprostinil	c.2549dupT (p.Met850Ilefs13) rs1560082927	Frameshift	9.21e-6 (no homo)	33	D	D	Zhu 2019^4^	P
							c.2227C>T (p.Arg743Cys)	Missense	absent	32	D	D	Zhu 2019^4^	LP
F3 I.1	F	22 months	alive	59*	34.3	Sildenafil, bosentan, treprostinil and digoxin	c.3079dupT (p.Trp1027Leufs9) rs746602775	Frameshift	absent	absent	---	--	no	P
							c.3685G>T (p.Glu1229) rs200914446	Nonsense	absent	26.4	---	---	no	P

Variant nomenclature according to transcript NM_001367549.1.

Deleteriousness predictions: CADD score>20: deleterious; SIFT or PolyPhen score=D: protein damaging.

ACMG class: LP: likely pathogenic; P: pathogenic.

*Non-responder to inhaled nitric oxide or oxygen. F1 II.1 and II.2 were not tested.

CADD, Combined Annotation Dependent Depletion; mPAP, mean pulmonary artery pressure; nk, not known; PAH, pulmonary arterial hypertension; PVRi, pulmonary vascular resistance index; SIFT, Sorting Intolerant from Tolerant.

### Family 2

Proband II.2 was born at 30 weeks’ gestation, weighing 790 g. After 2 months in the neonatal intensive care unit, he was discharged home. At age 6 months, he was taken to his primary care provider due to delayed developmental milestones and episodes of cyanosis. Echocardiography demonstrated severe PAH with RV pressure greater than 71 mm Hg. There was no congenital heart disease. RHC showed a mPAP of 34 mm Hg with a pulmonary vascular resistance (PVR) index of 11.8 Woods units that did not respond to oxygen or inhaled nitric oxide (iNO). Following a cardiac arrest during recovery from the procedure, he was supported by extracorporeal membrane oxygenation for 10 days. Despite extubation and maintenance therapy, he remained in critical condition and died of sepsis and PAH at age 11 months.

The proband’s sibling, II.4, was born at 37 weeks’ gestation. Echocardiography on the first day of life showed an estimated near systemic RV systolic pressure. At age 1 week, she developed persistent cyanotic episodes, with oxygen saturations in the 70 s and respiratory distress. RV pressure was observed to be suprasystemic by echocardiography. She was initially treated with inhaled epoprostenol and NO, vasopressors and mechanical ventilation. She then received extracorporeal membrane oxygenation for 5 days, due to acute decompensation. She subsequently responded to oxygen, diuretics, sildenafil, bosentan and treprostinil and was discharged at age 2 months. Follow-up echocardiography showed near systemic RV pressure and moderate RV hypertrophy. RHC at age 9 months showed a mPAP of 51 mm Hg with a PVR index of 11.8 Woods units, and these did not improve substantively with iNO and oxygen. Vasoreactivity testing showed no response until inhaled epoprostenol was added to 100% oxygen and iNO, perhaps indicating more refractory disease requiring engagement of both cyclic AMP and GMP pathways to maximise vasodilatation. She died at age 17 months, after contracting a respiratory infection. Both parents (I.1 and I.2) and two siblings (II.1 and II.3) remain healthy with no signs of PAH. The family is of Ashkenazi Jewish ancestry (table 1).

### Family 3

Proband II.3 presented with severe PAH and right heart failure at age 22 months. Echocardiography and chest CT angiogram established RV dilatation and hypertrophy with right atrial enlargement. RHC demonstrated suprasystemic pulmonary artery pressure, a PVR of 34.3 and a low cardiac index (CI 1.7) that was mildly responsive to oxygen and iNO (PVR decreased to 25.5). Given her severe presentation and pulmonary haemodynamics, balloon dilatation of her patent foramen ovale was performed. Triple therapy (subcutaneous remodulin, oral bosentan and oral sildenafil) was initiated for 1 year, but haemodynamic values were only modestly improved (PVR 12.5, CI 3.6), with a mild response to oxygen and iNO (PVR decreased to 9.8). Despite continued triple therapy, she re-presented with signs of right heart failure 6 months later. RHC showed suprasystemic pulmonary artery pressure and a PVR of 22 that was unresponsive to oxygen and iNO. An MRI demonstrated a RV ejection fraction of 49%. Given her malignant course that was refractory to therapy, a Potts shunt was performed via left thoracotomy. Three months after her Potts procedure, she has much improved exercise tolerance. Both parents (I.1 and I.2) and two siblings (II.1 and II.2) are asymptomatic, and her sibling II.2 had a normal echocardiogram. The family is of Anglo-European ancestry (table 1).

## Methods

### Patient recruitment

Family 1 was recruited for genetic analysis through the NIHR BioResource for Rare Diseases programme.^13^ Family 2 was enrolled into a genetic study of PAH reviewed by the Columbia University Irving Medical Center institutional review board. Family 3 was enrolled in a research protocol approved by the University of California San Francisco institutional review board. All family members who were genetically assessed provided informed consent for themselves or their children.

### Molecular genetic analysis

For family 1, DNA was extracted from whole blood samples for I.1, I.2 and II.2 and from a dried blood spot from the proband II.1 using the truXTRAC DBS Kit (Covaris). Parent–offspring trio (I.1, I.2 and II.2) whole genome sequencing was performed as described.^3^ For family 2, chromosomal microarray analysis and clinical exome sequencing were performed using blood-derived DNA from II.2 and II.4. Reanalysis of exome sequencing data screened 19 PAH risk genes^1 14^ (*ACVRL1*, *AQP1*, *ATP13A3*, *BMPR1B*, *BMPR2*, *CAV1*, *CD248*, *EFCABB4B*, *EIF2AK4*, *ENG*, *GDF2*, *KCNA5*, *KCNK3*, *KLF2*, *SMAD1*, *SMAD4*, *SMAD9*, *SOX17* and *TBX4*). Analysis of Family 3 proband II.1 employed targeted sequencing of 21 genes associated with PAH (*ABCC8*, *ACVRL1*, *AQP1*, *ATP13A3*, *BMPR1B*, *BMPR2*, *CAV1*, *EIF2AK4*, *ENG*, *FOXF1*, *GDF2*, *KCNA5*, *KCNK3*, *KLF2*, *NFU1*, *NOTCH3*, *RASA1*, *SMAD4*, *SMAD9*, *SOX17* and *TBX4*).

Family 1 samples were sequenced using Illumina HiSeq (2500 or X) technology, generating paired-end reads. All samples had a minimum read-depth coverage of 15× in ≥95% of targeted regions. Quality control, read mapping, variant calling and annotation were all conducted as described.^3^ Likely pathogenic variants were independently confirmed by Sanger sequencing. Families 2 and 3 underwent clinical sequencing by GeneDx^15^ or Blueprint Genetics, respectively. Population allele frequencies were obtained from the Genome Aggregation Database (gnomAD) V.2.1.1 (controls) database. In silico pathogenicity predictions were assessed using Combined Annotation Dependent Depletion score, Sorting Intolerant from Tolerant and PolyPhen-2.^16^ Additional family members were genotyped by Sanger sequencing.

## Results

### Family 1

**Figure 1:**
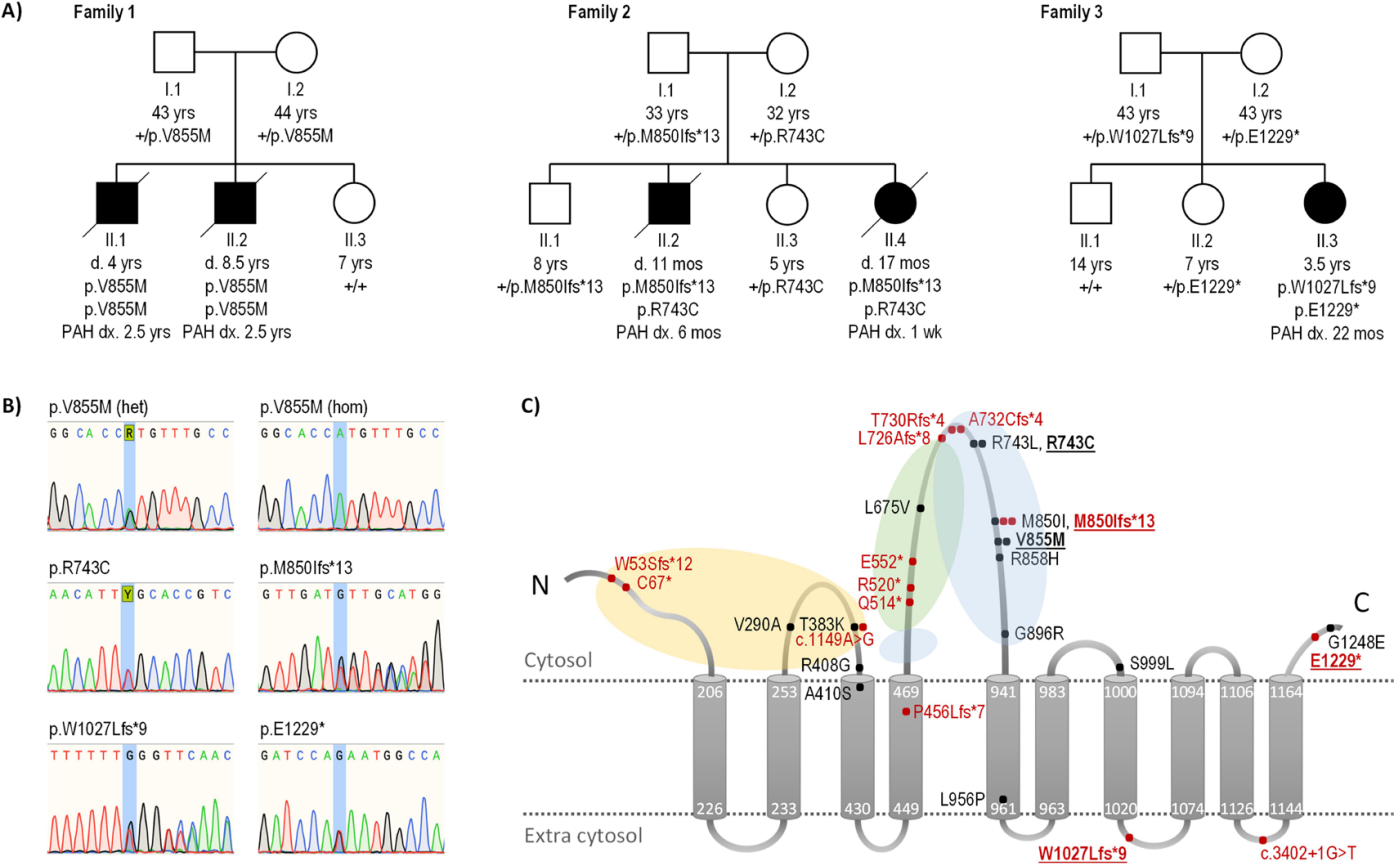
Rare deleterious ATP13A3 variants in biallelic and monoallelic PAH. (A) Family pedigrees for cases with early-onset, severe biallelic PAH. Filled boxes and circles indicate affected individuals. ATP13A3 mutation status is detailed for each individual. (B) Sequence chromatograms illustrating identified ATP13A3 variants in families 1–3. (C) ATP13A3 topology model with locations of variants identified in all PAH cases, both novel (bold typeface) and previously reported (Graf 2018, Zhu 2019, Wang, 2019, Lerche 2019, Gelinas 2020). Missense variants are in black, predicted truncating/splice variants in red. The number of filled circles at a variant location indicates the number of unrelated PAH cases identified with the variant. Yellow, green and blue shaded areas represent the actuator, nucleotide-binding and phosphorylation domains, respectively. Amino acids are numbered according to the longest transcript (NM_001367549.1; NP_001354478.1). +, wild type allele; d., age at death; dx., age at diagnosis; het, heterozygous; hom, homozygous; mos, months; PAH, pulmonary arterial hypertension.

Trio analysis identified a homozygous *ATP13A3* missense variant (NM_001367549.1: c.2563G>A; p.Val855Met) in affected child II.2, which was heterozygous in both parents (I.1 and I.2) (table 1). The variant is absent from gnomAD and a non-PAH European cohort (n=6385)^3^ and is predicted to be deleterious using in silico analysis (table 1). The deceased sibling (II.1) was homozygous for p.Val855Met and the unaffected sister (II.3) homozygous for the reference allele (figure 1A). The grandparents of the affected brothers are from nearby villages potentially indicating a common ancestor. The p.Val855Met variant resides in the likely kinase active cytoplasmic loop between the fourth and fifth transmembrane domains (figure 1B).^17^ Of note, a homozygous c.2563G>A substitution was recently reported in a Southern European cohort^14^ but variant cosegregation with affected relatives was not evaluated in this study.

### Family 2

Targeted reanalysis detected compound heterozygous *ATP13A3* variants in both affected children (II.2 and II.4): c.2227C>T; p.Arg743Cys and c.2549dupT; p.Met850Ilefs*13. Both parents (I.1 and I.2) and two unaffected siblings (II.1 and II.3) are heterozygous for one of the two variants (figure 1A) but remain asymptomatic. The p.Arg743Cys missense variant is absent from gnomAD and is predicted to be deleterious in silico (table 1). The variant resides in the same cytoplasmic loop as the family 1 variant (figure 1B), and a distinct monoallelic missense variant (c.2228G>T; p.Arg743Leu) impacting the same amino acid residue has been observed in an adult-onset IPAH case.^4^ *ATP13A3* is highly constrained for loss-of-function variants (pLoF=1).^18^ The p.Met850Ilefs*13 frameshift is extremely rare (allele frequency 9.21e-6, with no homozygotes observed in gnomAD) and has been reported in a monoallelic adult-onset IPAH case.^4^ Of note, the two allele counts reported in gnomAD are from the Ashkenazi Jewish population similar to our family.

### Family 3

Targeted screening identified compound heterozygous variants of *ATP13A3* in the affected child (II.1): c.3079dupT; p.Trp1027Leufs*9 and c.3685G>T; p.Glu1229* (table 1). Both parents (I.1 and I.2) and one unaffected sibling (II.2) are heterozygous for one of the two variants; another unaffected sibling (II.1) is homozygous for the reference allele (figure 1A). The c.3079dupT mutant transcript is likely degraded by nonsense-mediated decay (NMD). However, the nonsense variant, c.3685G>T, is located in the final exon of the gene and is therefore predicted to escape NMD^19^ leading to the production of an abnormal, potentially damaging protein product.

## Discussion

We report biallelic variants in *ATP13A3* associated with very early age of PAH onset and high mortality in three independent families. The five variants are extremely rare or absent from gnomAD and predicted to be deleterious missense or loss-of-function variants. Some of the variants have been reported for monoallelic adult-onset PAH. Therefore, the relatively young parents and siblings of the affected children, who are currently asymptomatic heterozygous carriers, are at increased risk of developing PAH later in life. These data support a novel paradigm of semi-dominant inheritance for *ATP13A3* in cPAH.

*ATP13A3* encodes a transmembrane cation transporter, which was recently shown to transport polyamines.^8^ Polyamines are small metabolites required for normal cell growth and proliferation, and elevated concentrations have been reported in multiple cancers and, more recently, PAH.^20 21^ *ATP13A3* is widely expressed in developing embryo and adult tissues,^22^ including pulmonary arterial smooth muscle cells (PASMC).^3^ Hypoxia, an environmental inducer of PAH, stimulates accumulation of spermine leading to increased PASMC proliferation in model systems. We hypothesise that *ATP13A3* variants predicted to alter transporter function disturb polyamine homeostasis. However, additional experiments are required to assess the disease mechanism.

Identification of PAH causal genes and variants provides critical information to begin to tailor approaches for treatment and prognosis. In vitro analysis of patient-derived loss-of-function *KCNK3* variants showed successful recovery of channel function in response to a phospholipase inhibitor, suggesting a novel therapeutic avenue for PAH.^1 2^ Similarly, experimental therapies targeting the polyamine transport system in cancer^23^ can now be tested in *ATP13A3*-targeted model systems relevant to PAH. An independent therapeutic approach includes adenoviral delivery of unmutated genes or molecular editing of mutated genes, in PAH relevant cell types, to restore normal gene expression or protein function, respectively. Finally, deep phenotyping and longitudinal follow-up of cases grouped by genetic diagnosis provides an opportunity to identify associated phenotypes to inform clinical care.

Emerging data from sequencing studies increases the utility of case-level genetic reanalysis. For family 2, the two affected children underwent extensive genetic testing without a diagnosis, but sequencing reanalysis facilitated the identification of biallelic variants in the newly identified *ATP13A3* gene. Our study indicates that genetic reanalysis may be an appropriate strategy to prioritise genetic diagnoses when preterm infants do not follow the usual trajectory expected from the degree of prematurity or when clinical diagnoses are ambiguous.

Identification of biallelic *ATP13A3* variants in three families with severe cPAH, in conjunction with recently identified monoallelic variants in adult-onset PAH, indicates semidominant inheritance for the *ATP13A3* gene. Biallelic inheritance indicates a dose-dependent threshold effect with implications for prognosis and treatment strategies. Taken together, these findings demonstrate the growing importance of comprehensive genetic analyses in well-powered cPAH populations.
